# Burrunan babble: acoustic characterization of the whistles and burst-pulse sounds of a critically endangered dolphin

**DOI:** 10.1098/rsos.241949

**Published:** 2025-07-16

**Authors:** Amber Crittenden, Christine Erbe, Amelia Street, Rebecca Wellard, Kate Robb

**Affiliations:** ^1^ Marine Mammal Foundation, Hampton East, Victoria 3188, Australia; ^2^ Centre for Marine Science and Technology, Curtin University, Perth, Western Australia, Australia; ^3^ Project ORCA, Perth, Western Australia, Australia

**Keywords:** Tursiops, vocal repertoire, whistle, burst-pulse sound, critically endangered

## Abstract

Understanding the vocal repertoire of delphinid species is essential for effective long-term studies. In this research, we present the first quantitative analysis of whistle and burst-pulse sound characteristics for the critically endangered Burrunan dolphin (*Tursiops australis*). Acoustic data were collected from the two known resident populations in Port Phillip Bay (PPB) and the Gippsland Lakes (GL), Victoria, Australia, between 2016 and 2023. Our analysis encompassed 12 973 signals, including 3401 whistles and 9572 burst-pulse sounds, drawn from nearly 21 h of recordings. We classified whistles into six contour classes, with upsweep (41.46%) and convex contours (24.64%) being the most prevalent. The fundamental frequency of whistles varied from 0.49 to 42.50 kHz, with durations ranging from 0.02 to 1.86 s. Burst-pulse sounds were categorized into four classes, with barks (71.74%) being the most common, displaying peak frequencies from 0.09 to 47.81 kHz and durations of 0.01–5.29 s. Significant variation in vocalization characteristics was observed between the two resident Burrunan dolphin populations, with PPB repertoire more restricted than GL. Variation in whistle repertoire composition between Burrunan and other *Tursiops* spp. was also found. This study provides crucial insights into the vocal characteristics of the Burrunan dolphin, which will inform conservation efforts.

## 1. Background

The Burrunan dolphin (*Tursiops australis*) is a recently described species of dolphin found in the coastal waters of southern and southeastern Australia [1]. The Burrunan dolphin exemplifies a small, coastal, genetically isolated species of odontocete, with only two known resident populations documented in southeastern Australia: Port Phillip Bay (PPB) and the Gippsland Lakes (GL), both located in the southern state of Victoria [1–3]. Genetic confirmation of the presence of this species in other locations along the southern Australian coastline exists, including southern Western Australia, South Australia and Tasmania [2,4,5]. While the species’ genetic status across its known distribution and the complex histories of these populations remain somewhat contentious [6,7], the populations in our study represent those in the original taxonomic description [1]. In our study region, Victoria, the known resident populations total fewer than 250 individuals [2]. Consequently, the species has been listed as critically endangered under the Victorian Flora and Fauna Guarantee Act, following International Union for Conservation of Nature (IUCN) Red List assessment criteria [8].

The locations in which these populations reside represent ecologically diverse yet anthropogenically impacted environments, with threats including vessels [9–11], lack of genetic diversity [2], fresh water skin disease [12] and toxicants [13]. This is of critical concern, as the Burrunan dolphin is a top-order predator within both environments and acts as a bioindicator for the overall health of the PPB and GL ecosystems [13–16]. In addition to their ecological value, they carry economic value, evident in the successful eco-tourism industries in both areas [17–20]. The value of the species has been formally recognized with the Burrunan dolphin listed as a ‘Natural Value’ of the state of Victoria [21] and dedicated conservation measures in place for the species, such as the designation of a marine sanctuary in PPB and the enforcement of strict approach-distance regulations [22]. For effective conservation management of the species, ongoing monitoring of its distribution, population dynamics and behaviour should be undertaken, growing our understanding of the species’ ecology, needs and threats. Passive acoustic monitoring (PAM) is one tool that can aid conservation monitoring.

Delphinid acoustic signals serve a variety of purposes, such as navigation, foraging and communication, including individual identification and interaction with conspecifics [23–27]. The global distribution and coastal occurrence of *Tursiops* spp. (bottlenose dolphins) has resulted in the extensive study of the overall repertoire of vocalizations produced, as well as the physical behaviours associated with vocalization types. Three generalized sound types are reported for the *Tursiops* spp. repertoire: whistles, clicks and burst-pulse sounds [26]. Whistles are frequency-modulated vocalizations associated with cohesive behaviours, such as social interactions, group-wide behavioural state changes and identity [25,28–35]. Echolocation clicks are broadband short-duration signals that enable the emitting animal to acquire information about its physical environment [36,37] and are therefore associated with navigation and prey capture [35]. Burst-pulse sounds are perhaps the least studied among *Tursiops* sound types and consist of rapid ‘packets’ of broadband short-duration pulses, with pulse-repetition rates exceeding those of echolocation click trains [38–41]. Burst-pulse sounds have been associated with aggressive behaviours [42,43] during defence or mating [44,45]. The identification of these sounds and the measurement of their specific characteristics can provide insight into the behaviour of the animal at the time of recording [35]. This is particularly beneficial when monitoring large areas over long time frames [46–49].

Boat-based visual observation programmes require the physical presence of researchers in the field (associated with various health, safety and environment risks), good weather and light [3,50,51]. Autonomous recorders associated with PAM studies remain deployed for weeks or months at a time and can overcome some of the limitations of visual observations but are limited to monitoring vocalizing marine species, such as *Tursiops* spp. [46,47,52–60]. However, effective PAM studies require the confident identification of the species-specific vocalizations [55,61]. The characterization of the Burrunan dolphin acoustic repertoire herein provides the baseline data required for PAM in studying this species.

To date, there is no formal documentation or description of the acoustic repertoire and potentially species-identifying characteristics of the Burrunan dolphin. We describe its whistles and burst-pulse sounds—with several classes of each of these sound types identified. We further compare these sound types across the two known resident populations of Burrunan dolphin, and to those of other known populations of *Tursiops* spp. from Australia, for similarities and any potential species-based differentiation. The characterization of the Burrunan dolphin vocal repertoire is expected to expand our capacity for the ongoing study of this species.

## 2. Material and methods

### 2.1. Study sites

**Figure 1.**
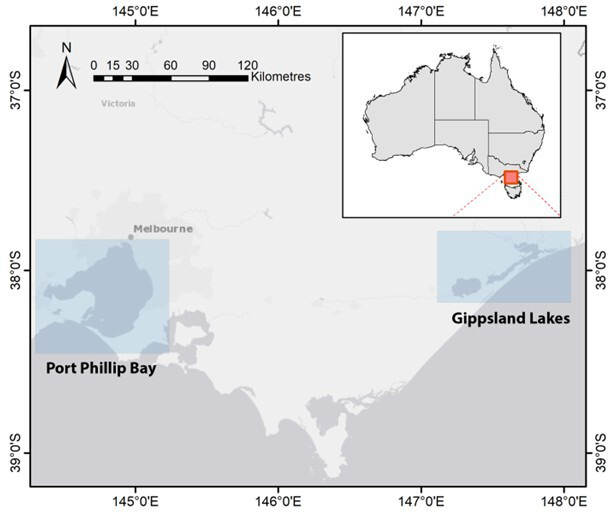
Map showing the two study sites, Port Phillip Bay (PPB) and the Gippsland Lakes (GL), on the southeastern coast of the state of Victoria, Australia.

This study examined the two Victorian resident populations of Burrunan dolphin in PPB and GL, southeastern Australia (figure 1). An embayment covering an area of approximately 1930 km^2^, PPB is subject to high recreational boat traffic, functions as a major commercial waterway and hosts the busiest shipping port in Australia as well as the country’s second largest metropolitan city, Melbourne [62]. It has an average depth of 13 m, shows relatively constant salinity and temperature, with oceanic water exchange via a single opening to Bass Strait at the southern end of the bay [62–64]. Conversely, GL is a series of large estuarine lakes, lagoons and marshes, covering approximately 600 km² along Victoria’s eastern coastline [65] with depths varying between 2 and 10 m across the three main lakes, averaging 4 m across the complex system. It is also the location of a large area of Ramsar wetlands, demonstrating its critical ecosystem role in global biodiversity [66]. The system is exposed to seasonal vessel traffic and commercial fisheries and shows large ecological variation from fresh water input from seven major rivers and saline water input from Bass Strait from a small (80 m) artificially maintained entrance [65,67,68].

### 2.2. Field data collection

Acoustic recordings were made opportunistically between 2016 and 2023 during boat-based surveys undertaken for Burrunan dolphin population study at the two field sites, PPB and GL. Handheld hydrophone recordings were collected with a CR-80-40 hydrophone (sensitivity: −166 ± 5 dB re 1 V µPa^−1^; frequency range: 7 Hz–80 kHz). Sound files were recorded on a H5 Zoom Handy Recorder, sampling frequency 96 kHz. Recordings were collected during dedicated focal follows of Burrunan dolphins, during daylight hours in calm water (less than 15 knots wind speed) where visibility was good (greater than 500 m). The hydrophone was deployed over the side of the research vessel to mid-water depth. Once dolphins were sighted, the predetermined survey transect was paused and global positioning system (GPS) location, environmental data, dolphin behavioural state, approximate group size and composition, and dorsal-fin identification photographs were taken [3]. A group of dolphins was defined as any dolphin in association with at least one other within a 100 m radius, moving in the same direction and often engaging in the same activity [69]. To minimize the possible effect of the research vessel on the dolphins’ behaviour, data were collected with the engine switched off and the boat positioned as close as possible to the observed group (within 100 m). Recordings were listened to in real time to determine whether vocalizations were being captured effectively. In the event the group moved too far away from the hydrophone and/or sound quality was reduced, the recording was aborted and the vessel repositioned maximizing potential for another opportune recording.

### 2.3. Data analysis

Acoustic recordings were downloaded and inspected both visually and aurally in Raven Pro 1.6.5 (Cornell Laboratory of Ornithology, Ithaca, NY, USA). Only recordings made during confirmed visual species sightings were included in analysis. Moreover, we excluded data from analyses when another distinguishable or indistinguishable odontocete species was observed near the pod. Spectrograms were created in windows of 1024 samples, Hamming windowed, with 50% overlap.

Whistles and burst-pulse sounds (hereafter referred to as ‘signals’) were visually graded based on their signal-to-noise ratio (SNR) to assess if they met the criteria for proceeding to measurement [70,71]. Specifically, the fundamental contour had to be discernible. Signals were categorized into: Grade 1 (‘Faint’ or ‘Poor’) if the signal was low in energy and therefore indistinct, the SNR was poor, the contour was partially obstructed by another sound (e.g. vessel noise) or the start and end points were not clearly visible; Grade 2 (‘Good’) if at least 80% of the signal was distinct and the start and end points were visible; Grade 3 (‘Strong’) if the signal was prominent and clear. Only signals meeting Grades 2 or 3 were selected for qualitative and quantitative analysis. Grade assessment and manual measurement of signals were conducted by three different observers, with verification of signal grade, measurement and class assignment conducted by the lead author for all whistles and burst-pulse sounds before inclusion in statistical analysis.

The contour of each whistle was qualitatively assessed through visual observation of spectrograms by three of the co-authors, each experienced observers. Each whistle was categorized into classes based on frequency modulation. While whistle classification based on visual characteristics has been determined reliable within teams of multiple observers [72], annotations were secondarily observed by the lead author to ensure there was no variation in the visual grading within the dataset. Throughout the literature there is a lack of standardization regarding how contours are grouped, with researchers ranging from ‘splitters’ to ‘lumpers’ [73]. Variability among authors was assessed by an inter-observer reliability test. Four of the study observers classed 120 randomly selected whistle contour images from the dataset to predefined classes. A Fleiss’ Kappa test was performed in MATLAB (v. R2021b; The MathsWorks Inc., Natick, MA, USA) to determine the level of agreement.

Whistles of changing contours were assigned the contour class that accounted for at least 90% of the duration of the vocalization. Whistles were sometimes recorded at fluctuating received level (i.e. the SNR changed over the course of a contour) resulting in sections of contours with varying degrees of clarity. Weak sections and gaps that did not exceed 10% of the duration of the fundamental frequency (assessed through visual approximation) were allowed. Gaps longer than 10% of the fundamental frequency duration were deemed to indicate the end of one signal and the beginning of another, leading to these whistles being measured individually. Identification of signature whistles and multi-looped whistles was excluded from this study, though they are believed to be present within this dataset and worthy of further study [73–75]. The fundamental frequency for each whistle was measured, with the presence of overtones noted but not included in contour measurement.

Burst-pulse sounds were also visually and aurally inspected and sorted by the observers into classes. These classes were later cross-referenced with burst-pulse sound classes defined in the literature to determine if they had been previously identified in other delphinid species. The degree of tonality, clarity of tonal contours and bandwidth, when observed using a 1024-point fast Fourier transform (FFT), were considered for class assessment. For burst-pulse sounds that occurred in trains (rhythmic repetitions of the same signal), the inter-pulse interval (IPI), train duration and the number of signals per train were computed. A train was determined to be complete at the point in time when the IPI exceeded double the median IPI. If the sound being observed did not fit into one of the types or classes presented in this study, it was excluded from analysis.

**Figure 2.**
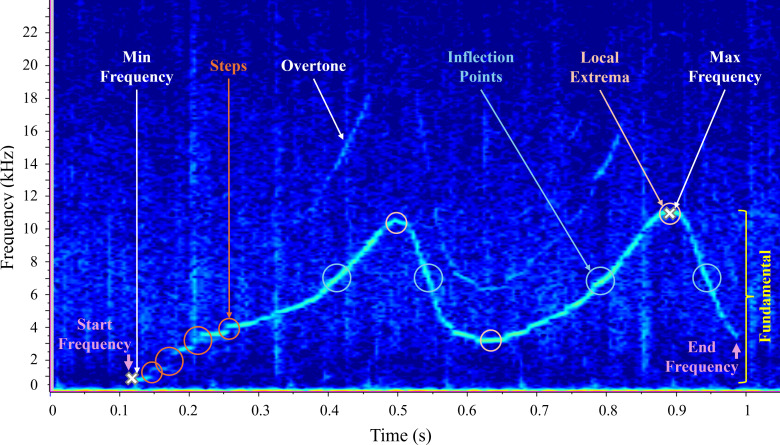
Spectrogram of a whistle contour illustrating parameters considered in this study. Sampling frequency = 96 kHz, number of fast Fourier transforms (NFFT) = 1024 and Hamming window, 50% overlap.

**Figure 3.**
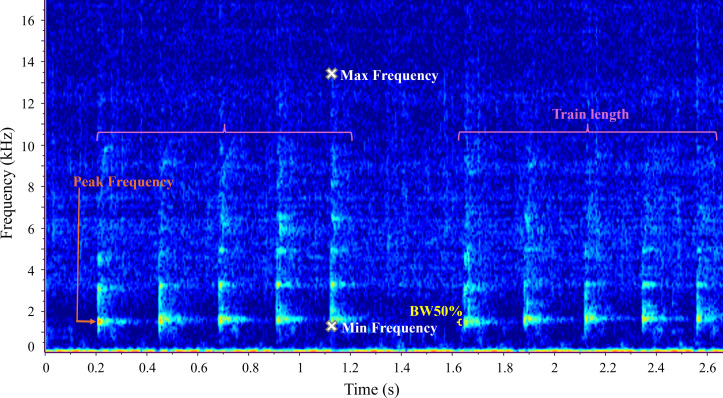
Spectrogram of two burst-pulse sound (class = bark) trains. Sampling frequency = 96 kHz, NFFT = 1024 and Hamming window, 50% overlap.

The complete list and definitions of 11 measured parameters can be found in table 1. The presence of overtones and number of steps informed whistle characterization, while peak frequency, 50% bandwidth and train length informed burst-pulse characterization. All parameters described in table 1 were measured in Raven Pro selection tables, with start and end frequency taken from the ‘peak frequency contour’ measurement, and duration, frequency range and train length calculated in Excel. Example spectrograms are shown in figure 2 (whistle) and figure 3 (burst-pulse sound).

**Table 1.** Complete list of whistle and burst-pulse sound parameters measured in Raven Pro and included in vocal repertoire analysis.

parameter	definition	units	signal
start frequency (*F*_start_)	the frequency at which the fundamental frequency began	kilohertz (kHz)	whistles
end frequency (*F*_end_)	the frequency at which the fundamental frequency ended	kilohertz (kHz)	whistles
minimum frequency (*F*_min_)	the lowest frequency present in a signal	kilohertz (kHz)	whistles, burst-pulse sounds
maximum frequency (*F*_max_)	the highest frequency present in a signal	kilohertz (kHz)	whistles, burst-pulse sounds
frequency range (*F*_range_)	the frequency range covered by the fundamental frequency (F_max_ – F_min_)	kilohertz (kHz)	whistles, burst-pulse sounds
duration	the length of time for which the signal was emitted	seconds (s)	whistles, burst-pulse sounds
presence of overtones	whether the fundamental frequency was accompanied by overtones (harmonic contours above the fundamental frequency), marked as binary present (1) or absent (0)	binary	whistles
number of steps	the number of breaks in a continuous whistle contour	manual count	whistles
peak frequency (*F*_peak_)	the frequency at which the burst-pulse sound exhibited the greatest energy	hertz (Hz)	burst-pulse sounds
50% bandwidth (BW 50%)	bandwidth about *F*_peak_, containing 50% of the signal energy	hertz (Hz)	burst-pulse sounds
train length (TL)	number of repeated signals within a train	no. of repetitions	burst-pulse sounds (barks and squeaks)

Histograms of the various whistle parameters were generated in Excel to show their frequency of occurrence and statistical distribution. A one-way multivariate analysis of variance (MANOVA) using a repeated measure model was conducted for each of the vocalization types (whistles and burst-pulse sounds) to determine whether vocalization characteristics (*F*_start_, *F*_end_, *F*_min_, *F*_max_, *F*_range_, duration, overtone presence, number of steps, *F*_peak_ and 50% bandwidth) differed significantly between populations (PPB and GL). Additional post hoc univariate analyses of variance (ANOVA) were conducted to reveal measurement parameters with significant difference between populations. A stacked bar graph representing the per cent occurrence of contour types within the Burrunan vocal repertoire and those of *Tursiops* spp. populations elsewhere in Australia (i.e. Fremantle [71,74], Bunbury [76,77] and Shark Bay [30,76], all located in Western Australia, and off the east coast in Moreton Bay, Queensland and Byron Bay, New South Wales [76]) was generated in Excel to compare whistle class composition across species and populations.

## 3. Results

Vocal repertoire analysis was conducted on a total of 20 h and 45 min of handheld hydrophone recordings collected in the presence of pods of dolphins, visually confirmed to be Burrunan dolphins. Recordings were collected during 110 unique dolphin pod sightings, with a minimum and maximum duration of approximately 30 min and 4 h, respectively, across 78 individual survey days between 2016 and 2023 over both study sites. From this dataset, a total of 3401 whistles and 9572 burst-pulse sounds that met the inclusion criteria were identified and analysed. The inter-observer reliability test determined perfect non-accidental agreement (Fleiss_κ = 0.84, confidence interval = 0.83−0.84, *p* < 0.001) between observers when assigning whistle contour class based solely on visual characteristics.

### 3.1. Whistles

**Figure 4.**
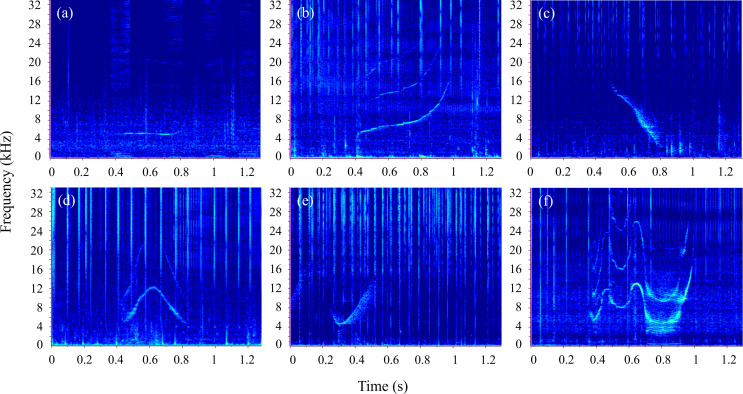
Spectrograms of the six whistle classes described: (a) constant, (b) upsweep, (c) downsweep, (d) convex, (e) concave and (f) sine. Sampling frequency = 96 kHz, NFFT = 1024 and Hamming window, 50% overlap.

Based on their fundamental contours, whistles were grouped into six classes: ‘constant’ (whistles with a mostly flat contour, for which the slope was less than 1000 Hz s^−1^), ‘upsweep’ (a monotonic increase in frequency), ‘downsweep’ (a monotonic decrease in frequency), ‘convex’ (inverted-U shape, i.e. upsweep followed by a local maximum, then downsweep), ‘concave’ (U-shaped contour, i.e. downsweep followed by a local minimum, then upsweep) and ‘sine’ (whistles with at least two extrema—local minima or maxima—and at least one inflection point, where the curvature changed, see [78] for definitions) (figure 4).

**Figure 5.**
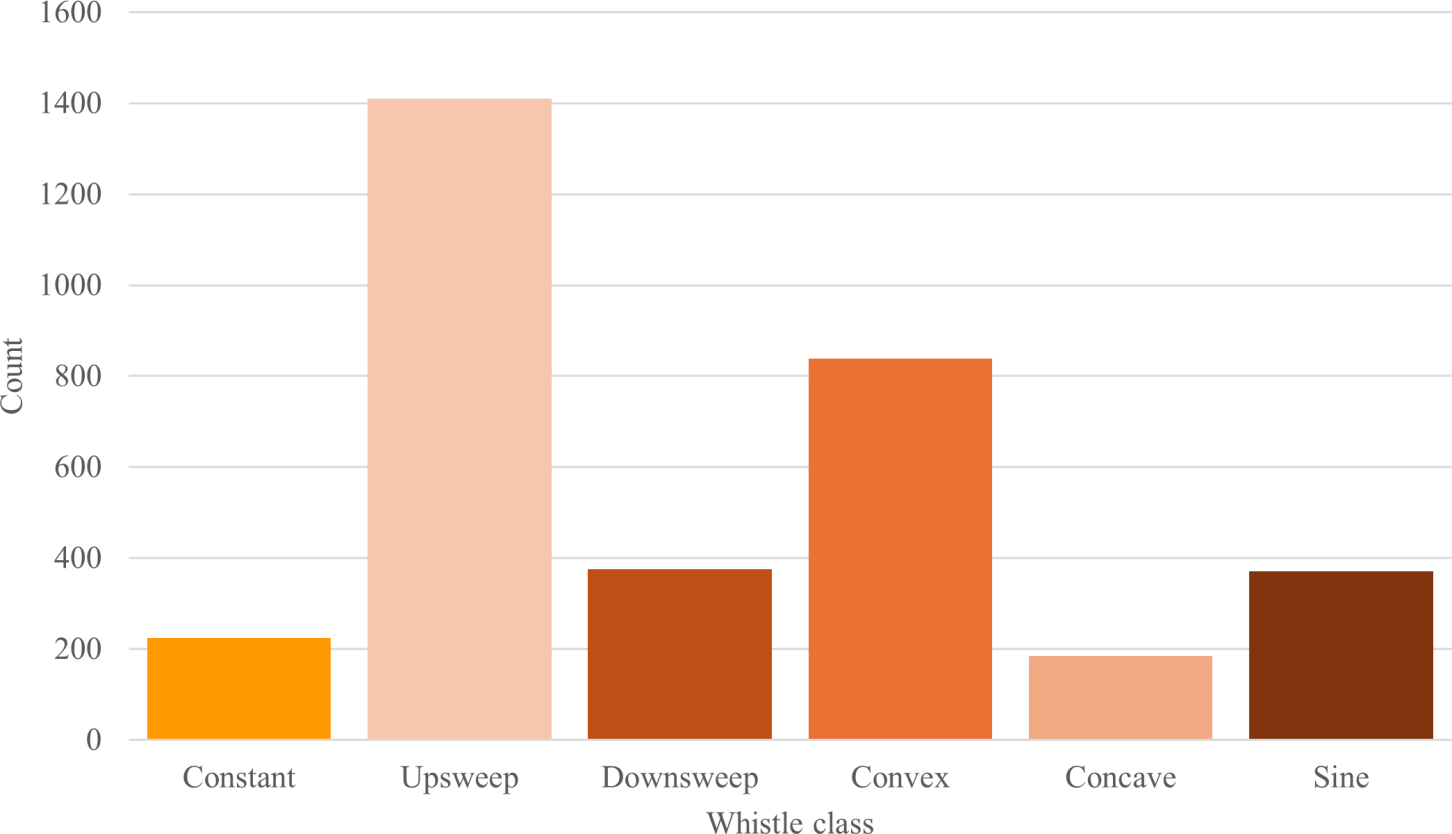
Histogram of the classes of Burrunan dolphin whistles measured at both study sites: PPB and GL, Victoria, Australia.

Of the 3401 whistles analysed, upsweeps were the most prevalent accounting for 41.46% (*n* = 1410), followed by convex (24.64%, *n* = 838), downsweep (11.03%, *n* = 375), sine (10.88%, *n* = 370), constant (6.59%, *n* = 224) and last, concave whistles (5.41%, *n* = 184; figure 5).

The fundamental frequencies spanned a frequency range from 0.49 to 42.50 kHz and duration of 0.02–1.86 s (table 2). Out of the 3401 whistles, 26.45% (*n* = 909) had steps. Whistles were almost equally likely to be with or without overtones, with 44.34% (*n* = 1524) of whistles showing the presence of overtones. Histograms showed that the duration of whistles and the frequency parameters (*F*_start_, *F*_end_, *F*_min_ and *F*_max_) was Poisson distributed, skewing towards lower values (see electronic supplementary material).

**Table 2.** Descriptive statistics, including mean, median, mode, standard deviation (s.d.) and ranges for the parameters measured of Burrunan dolphin whistles at both study sites: PPB and GL, Victoria, Australia.

parameters	mean	median	mode	s.d.	minimum	maximum
*F*_start_ (kHz)	8.22	6.94	5.63	5.28	0.75	42.09
*F*_end_ (kHz)	9.34	8.25	6.19	5.19	0.75	41.34
*F*_min_ (kHz)	6.90	5.59	5.22	4.87	0.49	37.37
*F*_max_ (kHz)	12.40	12.03	10.61	5.21	0.97	42.50
*F*_range_ (kHz)	5.50	5.06	3.97	3.56	0.16	19.45
duration (s)	0.28	0.23	0.43	0.22	0.02	1.86
number of steps	1.40	0	0	3.16	0	32.00

### 3.2. Burst-pulse sounds

**Figure 6.**
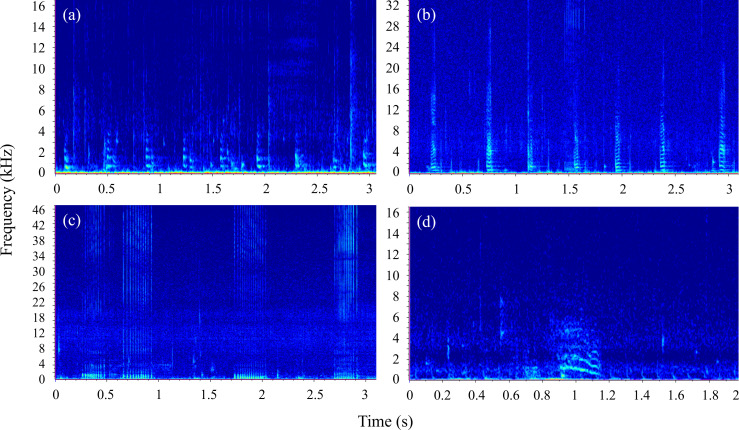
Spectrograms of the four burst-pulse sound classes described: (a) bark, (b) squeak, (c) creak and (d) moan. Sampling frequency = 96 kHz, NFFT = 1024 and Hamming window, 50% overlap.

Four classes of burst-pulse sounds were identified for the Burrunan dolphin following visual and aural screening: ‘bark’ (pulsed sound of distinct peak frequency below 2 kHz, emitted in long trains, often possessing energy in higher frequency bands), ‘squeak’ (rapidly pulsed sound appearing in spectrograms as tonal with many overtones, emitted in short trains), ‘creak’ (packet of low-frequency pulsed sounds emitted in short trains, often associated with broadband echolocation clicks) and ‘moan’ (low-frequency pulsed sounds of varying spectro-temporal characteristics but sharing a ‘moan’, ‘grunt’ or ‘groan’ aural quality) (figure 6). The classes were named for their aural characteristics.

**Figure 7.**
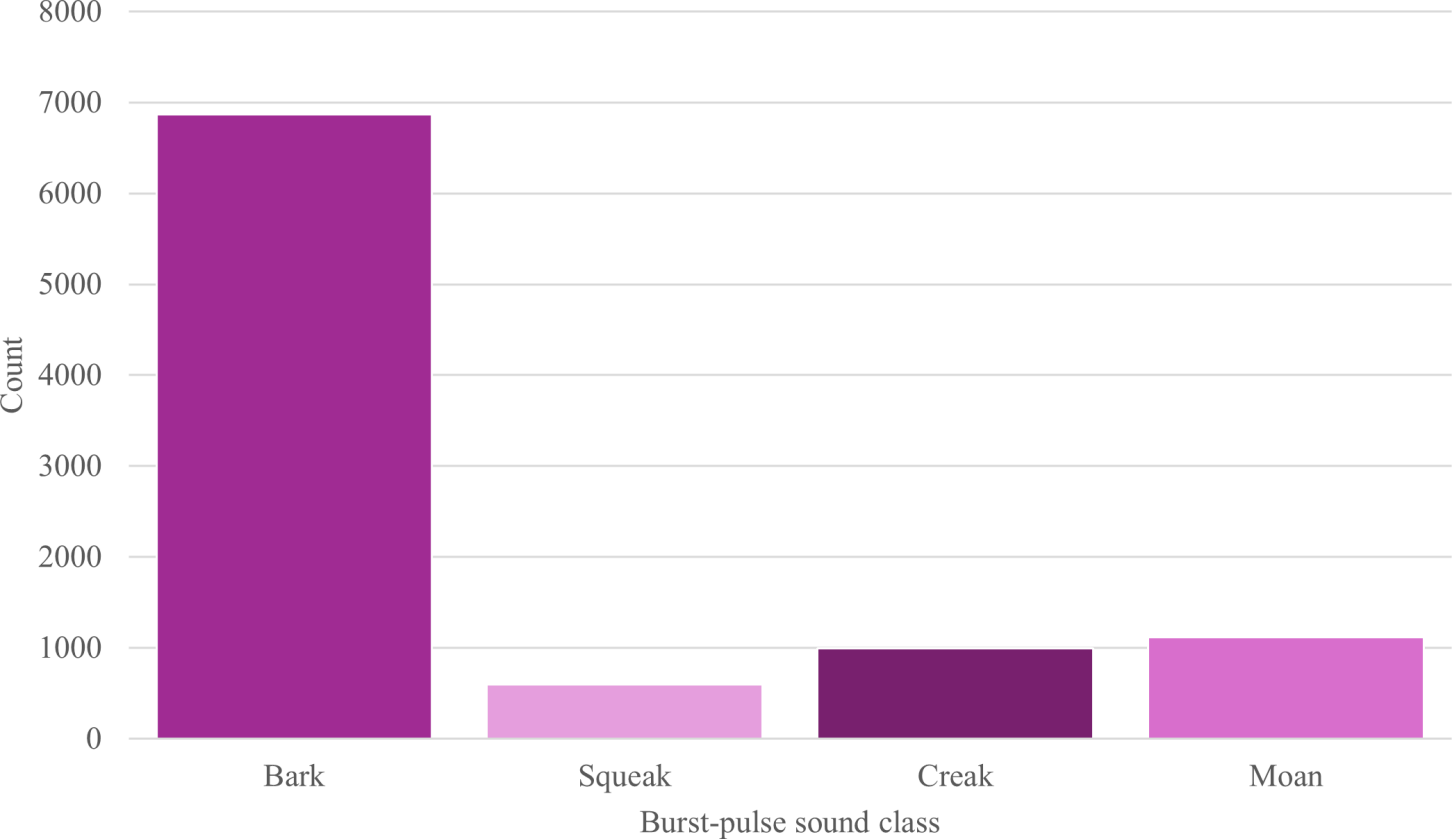
Histogram of the classes of Burrunan dolphin burst-pulse sounds measured at both study sites: PPB and GL, Victoria, Australia.

Of the 9572 burst-pulse sounds analysed, barks were the most prevalent class accounting for 71.74% (*n* = 6867), followed by moans (11.67%, *n* = 1117), creaks (10.37%, *n* = 993) and, last, squeaks (6.22%, *n* = 595; figure 7).

Given the variability in spectro-temporal characteristics and aural/visual qualities of the burst-pulse sound classes, as well as the lack of standardization of naming convention in the literature, each of the four burst-pulse sound classes was explored individually and is below described.

#### 3.2.1. Barks

Barks have been characterized here by the duration of the individual pulse events. Barks exhibited a peak frequency within the range of 0.19–6.94 kHz and duration of 0.01–0.26 s. The 50% bandwidth of barks ranged up to 7.13 kHz, with trains lasting a mean of 10.94 repetitions (see electronic supplementary material).

#### 3.2.2. Squeaks

Squeaks are rapidly pulsed and so appear tonal in spectrograms, with many overtones. Squeaks were found to exhibit a peak frequency within the range of 0.19–36.38 kHz and duration of 0.01–0.26 s. The 50% bandwidth of Burrunan dolphin squeaks ranged from 0.19 to 19.50 kHz. Squeak trains had a mean length of 4.48 repetitions (see electronic supplementary material).

#### 3.2.3. Creaks

Creaks refer to trains of both narrowband and broadband, short-duration, unmodulated distinct pulses when observed using 1024-point FFT, with a closely spaced IPI and low minimum frequency. They have an average duration of approximately 0.5 s and possess an aural quality of a creaking door or squealing gate. Creaks had a peak frequency within the range of 0.09–47.81 kHz and duration of 0.06–5.29 s. The 50% bandwidth of Burrunan dolphin creaks ranged between 0.19 and 42.64 kHz (see electronic supplementary material). Some creaks had energy all the way to the Nyquist frequency of 48 000 Hz, which, therefore, is the maximum frequency we report.

#### 3.2.4. Moans

Moans refer to low-frequency packets of pulses that, when observed using 1024-point FFT, resemble stacked, tonal contours. Moans had a peak frequency within the range of 0.19–18.19 kHz and duration of 0.02 –3.91 s. The 50% bandwidth of Burrunan dolphin moans ranged between 0.19 and 6.94 kHz (see electronic supplementary material).

### 3.3. Victorian populations: repertoire comparison

**Figure 8.**
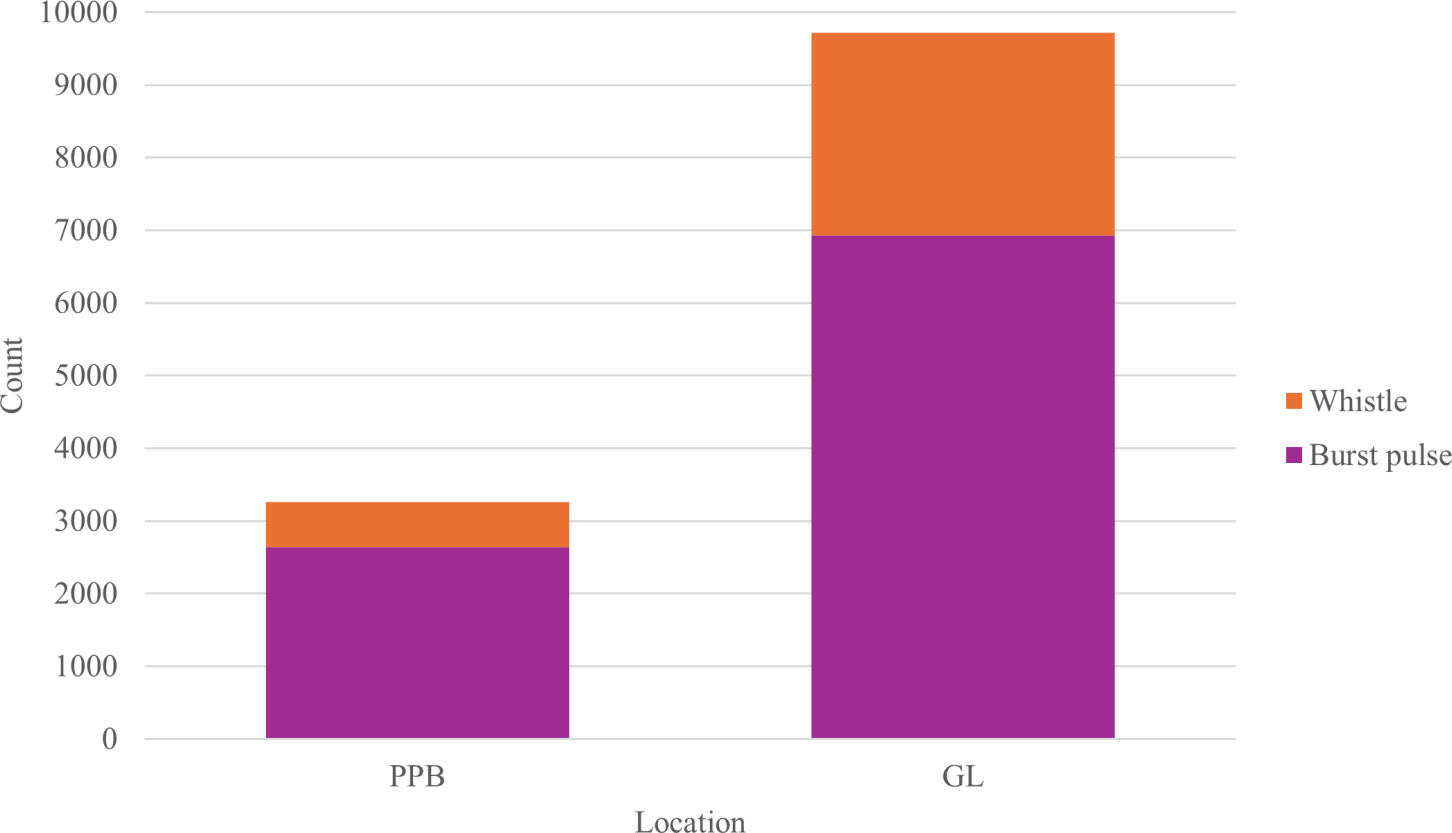
Comparison of vocal repertoire composition by signal type (whistles and burst-pulse sounds) in the two Burrunan dolphin populations: PPB and GL, Victoria, Australia.

Of the 3401 whistles and 9572 burst-pulse sounds characterized, 25.12% (*n* = 3259) were recorded in PPB and 74.88% (*n* = 9714) in GL (figure 8). This, however, may be reflective of the discrepancy in recording hours, with 22.19% (4 h 36 min) of total recording collected in PPB and 77.81% (16 h 8 min) collected in GL. Normalized by recording effort, PPB recorded approximately 134 whistles and 575 burst-pulse sounds per hour and GL recorded approximately 173 whistles and 429 burst-pulse sounds per hour.

Both populations displayed a higher proportion of burst-pulse sounds (than whistles), with 81.10% (*n* = 2643) of signals in PPB and 71.33% (*n* = 6929) of signals in GL being burst-pulse sounds (figure 8).

**Figure 9.**
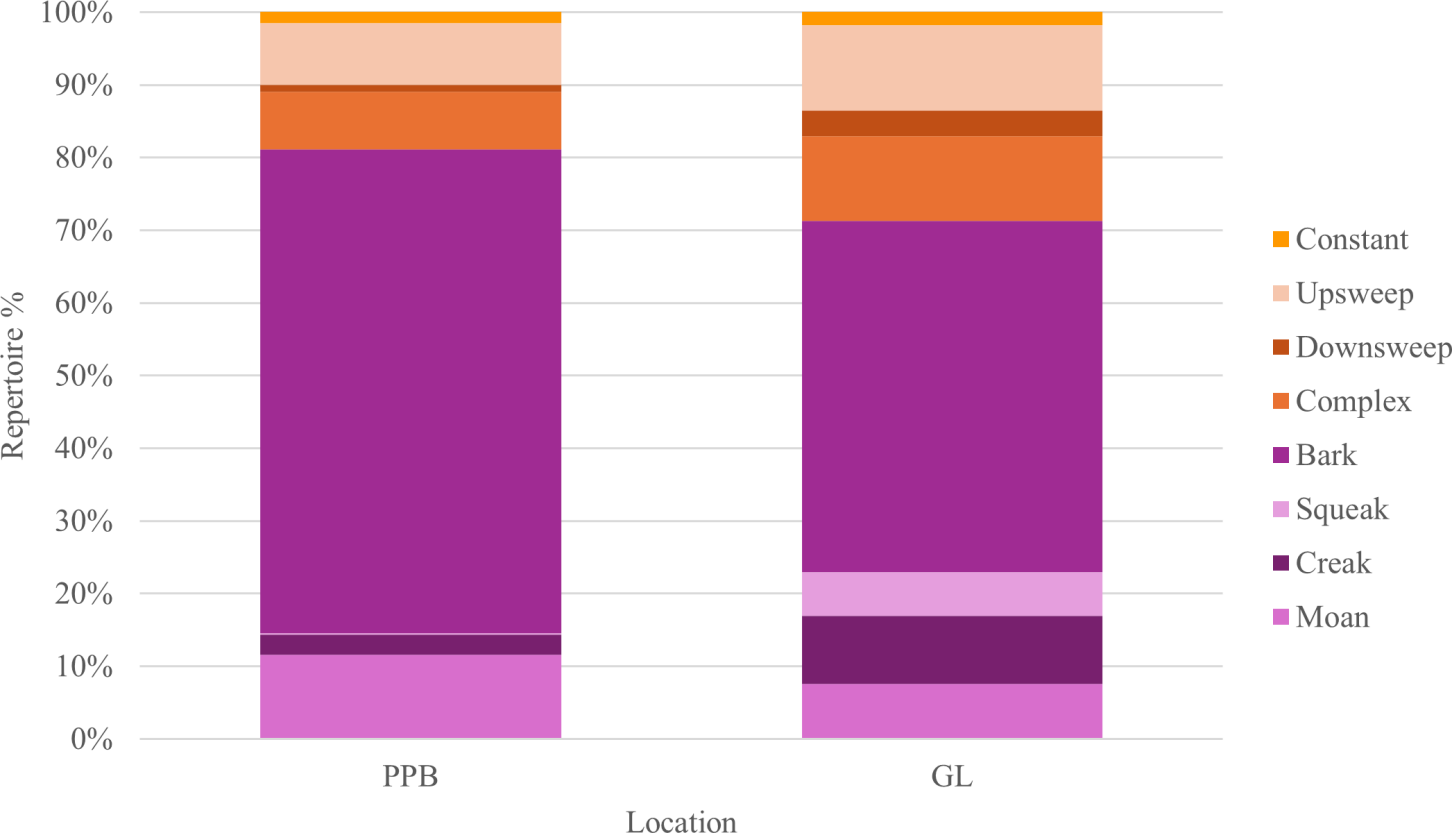
Comparison of vocal repertoire composition by signal class (whistles: constant, upsweep, downsweep and complex; and burst-pulse sounds: bark, squeak, creak and moan) in the two Burrunan dolphin populations: PPB and GL, Victoria, Australia.

Barks were the largest represented class of the repertoire of both populations, comprising 66.55% (*n* = 2169) and 48.36% (*n* = 4698) in PPB and GL, respectively (figure 9). Whistle contours classed in the Burrunan dolphin repertoire as convex, concave and sine have here been grouped into a ‘complex’ category to allow comparison with the other studies with more limited contour classes. The upsweep whistle in both locations (PPB: 8.47%, *n* = 276; GL: 11.67%, *n* = 1134) represented the greatest whistle class contributor (figure 9).

Overall, mean and median values for all measured whistle parameters were very similar between the PPB and GL repertoires. Of note, standard deviations and maximum values observed in *F*_start_, *F*_end_, *F*_min_ and *F*_max_ were greater in the GL whistle repertoire. GL showed a greater likelihood for whistle contours to display overtones and also exhibited a greater number of steps (table 3).

**Table 3.** Descriptive statistics of measured parameters for whistles recorded from PPB and GL Burrunan dolphins.

location	parameters	mean	median	mode	s.d.	min	max
PPB	*F*_start_ (kHz)	8.06	7.88	8.25	2.97	1.31	19.69
	*F*_end_ (kHz)	9.01	8.63	8.63	3.11	0.94	20.06
	*F*_min_ (kHz)	6.94	6.94	7.92	2.76	0.61	19.43
	*F*_max_ (kHz)	12.42	12.28	9.93	3.47	1.37	29.44
	*F*_range_ (kHz)	5.48	5.37	3.35	3.54	0.16	16.35
	duration (s)	0.31	0.25	0.39	0.24	0.02	1.32
	no. steps	0.96	0	0	2.36	0	17.00
GL	*F*_start_ (Hz)	8.30	6.75	5.63	5.68	0.75	42.09
	*F*_end_ (Hz)	9.48	8.25	6.19	5.54	0.75	41.34
	*F*_min_ (kHz)	6.93	5.43	5.22	5.24	0.49	37.37
	*F*_max_ (kHz)	12.40	11.97	10.61	5.53	0.97	42.50
	*F*_range_ (kHz)	5.47	5	1.17	3.56	0.25	19.45
	duration (s)	0.27	0.21	0.43	0.22	0.02	1.86
	no. steps	1.50	0	0	3.30	0	32.00

Mean and median values of the frequencies of burst-pulse sounds were higher in the GL population. This may be due to the greater number of creaks present and measured in GL when compared with PPB (*n* = 904 and *n* = 89, respectively), as creaks cover a wider bandwidth than other burst-pulse sound classes. This is also reflected in the variation of *F*_range_ standard deviation in each location (PPB s.d. = 5.82, GL s.d. = 13.23; table 4). The shortest signal duration was the same in both location (*n* = 0.01 s), but PPB exhibited shorter maximum durations (*n* = 3.21 s) than GL (*n* = 5.29 s). Peak frequency and 50% bandwidth had closer similarity between locations (table 4).

**Table 4.** Descriptive statistics of measured parameters for burst-pulse sounds recorded in PPB and GL Burrunan dolphins.

location	parameters	mean	median	mode	SD	min	max
PPB	*F*_min_ (kHz)	0.33	0.29	0.23	0.16	0.03	3.05
	*F*_max_ (kHz)	2.62	1.68	48	5.84	0.43	48
	*F*_range_ (kHz)	2.29	1.35	1.13	5.82	0.23	47.84
	duration (s)	0.10	0.06	0.07	0.18	0.01	3.21
	*F*_peak_ (kHz)	0.75	0.56	0.56	2.08	0.19	47.81
	BW 50% (kHz)	0.58	0.19	0.19	2.36	0.19	37.50
GL	*F*_min_ (kHz)	0.63	0.54	0.57	0.58	0.03	28.32
	*F*_max_ (kHz)	9.90	4.69	48	13.20	0.58	48
	*F*_range_ (kHz)	9.27	4.08	47.81	13.23	0.31	47.96
	duration (s)	0.15	0.03	0.03	0.37	0.01	5.29
	*F*_peak_ (kHz)	2.24	0.94	0.94	6.12	0.09	47.06
	BW 50% (kHz)	2.33	0.56	0.19	5.61	0.09	42.94
	*F*_min_ (kHz)	0.63	0.54	0.57	0.58	0.03	28.32

Results of the MANOVA showed that whistles and burst-pulse sounds had a statistically significant multivariate effect dependent on location (Pillai’s trace = 0.034 for whistles and 0.136 for burst-pulse sounds, *F*-value 17 and 214, *R*^2^ 0.03 and 0.14, respectively, *p* < 0.001). Thus, the PPB and GL Burrunan dolphin populations have significantly different vocal repertoires, despite populations comprising conspecifics, suggesting factors other than genetic speciation affect vocal repertoire composition.

The post hoc ANOVA tests revealed *F*_start_, *F*_end_, duration, presence of overtones and number of steps were significantly different in whistles across populations, while all measured parameters of burst-pulse sounds indicated statistically significant difference across populations.

### 3.4. Burrunan dolphin versus Australian *Tursiops* spp. whistle repertoire

**Figure 10.**
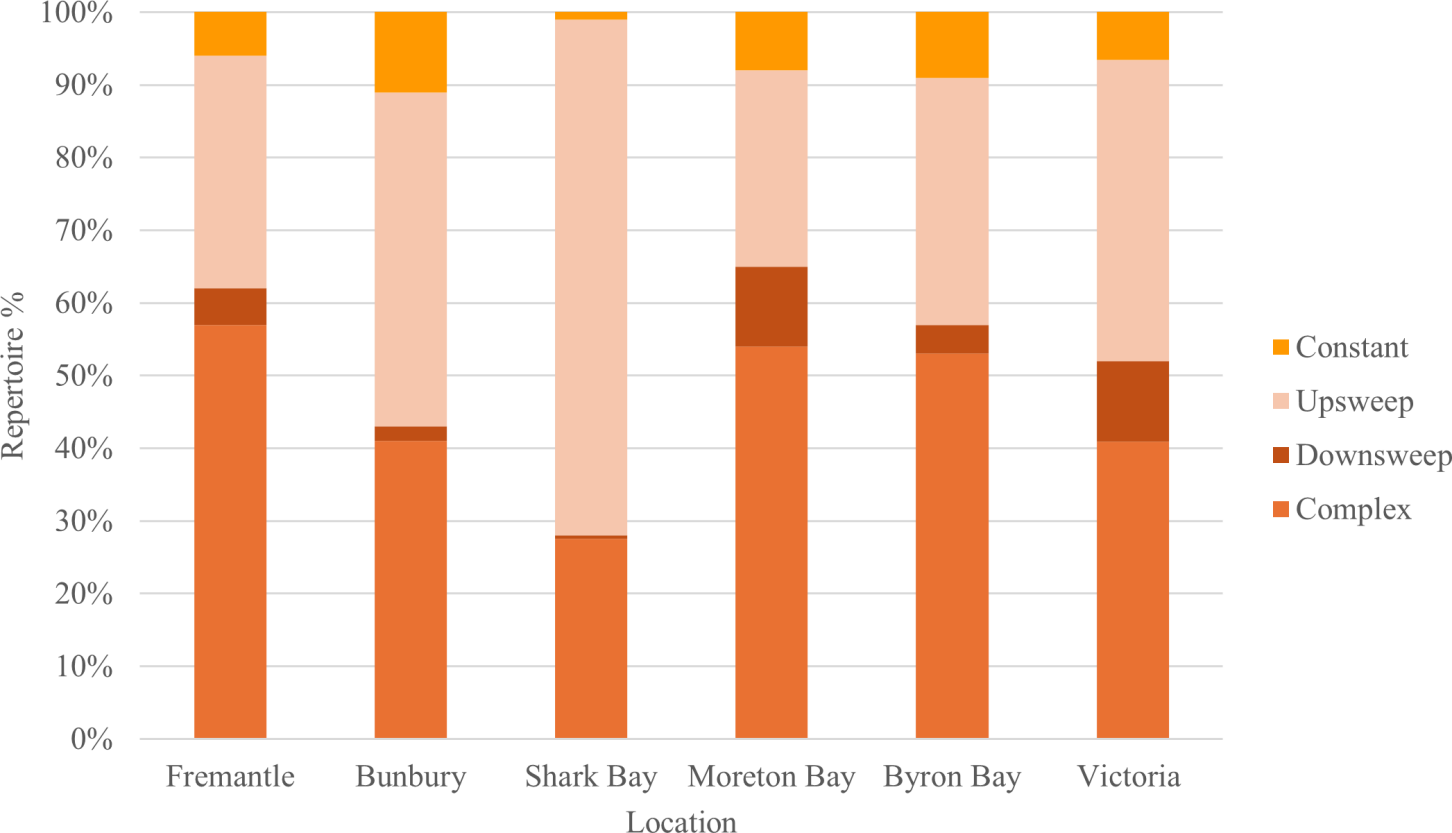
Comparison of classes as a proportion of whistle vocal repertoire for Tursiops spp. populations at Fremantle, Bunbury and Shark Bay on the Western Australian coast, Moreton Bay and Byron Bay on the east coast of Australia, and Victoria (Burrunan dolphin).

The comparison of the Burrunan dolphin whistle repertoire composition with that of other resident *Tursiops* spp. populations along the Australian coastline is illustrated in figure 10.

The proportion of contour classes within each whistle repertoire varied across the populations. The upsweep class represented the largest proportion of the whistle repertoires of the Bunbury (*n* = 46%), Shark Bay (*n* = 71%) and Victorian (*n* = 41.1%) populations, whereas the complex class represented the largest proportion of the Fremantle (*n* = 57%), Moreton Bay (*n* = 54%) and Byron Bay (*n* = 53%) populations (figure 10). Interestingly, there was similarity in overall whistle repertoire composition across different populations and coastlines. The west-coast Fremantle population whistle repertoire composition was most similar to those of the east-coast Moreton Bay and Byron Bay populations, and the west-coast Bunbury population showed closest similarity to the east-coast Victorian population whistle repertoire composition (figure 10).

## 4. Discussion

This study characterized the vocal repertoire of the Burrunan dolphin, captured in handheld hydrophone data collected across the two known resident populations in Victoria: PPB and the GL. The vocal repertoire comprised six putative whistle classes (constant, upsweep, downsweep, convex, concave and sine) and four notable yet poorly defined burst-pulse sound classes (squeaks, barks, creaks and moans). Similar vocalization characteristics were observed between the PPB and GL populations, with some variations in standard deviations of whistle frequency characteristics, whistle contour step count and burst-pulse class occurrence. Similarities in whistle repertoire were seen in several *Tursiops* spp. populations across Australia, with the whistle repertoire composition of the Victorian Burrunan dolphin most similar to that of the resident population of Indo-Pacific bottlenose dolphins in Bunbury, WA.

### 4.1. Whistles

Whistles are believed to be associated with a variety of behavioural contexts [79] but have been most commonly associated with social and cooperative behaviours. Upsweeps are commonly found to be the most frequent whistle class in *Tursiops* whistle repertoire. Like the Burrunan dolphin, high proportions of upsweep whistles have also been reported in *Tursiops* spp. In Shark Bay on Australia’s western coastline [30,76], Mexico’s Revillagigedo Archipelago [31] and in the Mediterranean Sea off the shores of Italy [80], among other *Tursiops* populations, indicating their significance as a vocal repertoire element and suggesting the upsweep whistle may be emitted in a variety of contexts. The upsweep is believed to be a vital element of communication, serving individual social interaction and group cohesion during cooperative behaviours such as feeding [24,80,81]. Upsweeps have also been documented in common bottlenose dolphins in biphonic emissions, whereby the upsweep is accompanied simultaneously by a second whistle or burst-pulse sound produced by the same animal [82]. These upsweep–burst-pulse biphonic combinations have been documented during trawl-fishing [83] suggesting the presence of these biphonics may be an indicator of stress. Multi-whistle biphonics have been recorded in social contexts [82], highlighting the flexible function of the upsweep whistle.

Specific features of fundamental whistle frequencies may be impacted by many drivers. Whistle production and fundamental frequency characteristics are found to be highly influenced by ambient noise. High levels of whistle stereotypy within acoustically complex environments [84,85], as well as simplification of whistle structure in noisy environments [86,87], are well documented in *Tursiops* spp. across the globe. That is, soundscapes containing higher numbers of sound sources are associated with more restricted delphinid whistle repertoires. One sound source that has been identified as a significant driver for whistle characteristics is vessel noise. Bottlenose dolphins are known to shift the bandwidths over which they emit whistles in the presence of vessel noise [88,89]. This deviation in typical fundamental frequency characteristics is believed to counteract the masking effect of propeller cavitation noise. Standard deviations in the frequency-related parameters of the Burrunan dolphin whistle repertoire were all approximately 4 kHz, indicating large variation in start, end, minimum and maximum fundamental frequencies of whistles. In this way, consideration of the large variability of the Burrunan dolphin whistle repertoire may indicate a high level of complexity within the acoustic habitats of PPB and GL.

### 4.2. Burst-pulse sounds

The literature suggests that *Tursiops* spp. produce more whistles than burst-pulse sounds. For example, burst-pulse sounds were only detected in 12% of acoustic encounters with common bottlenose dolphins in Walvis Bay, Namibia [42], comprised 12.3% of overall repertoire in common bottlenose dolphins in the Gulf of Mexico [43], occurred in 39.3% of recordings of bottlenose dolphins in Brisbane, Australia [90] and were present in 47% of recorded events in captive common bottlenose dolphins in France [91]. With approximately 60% representation of burst-pulse sounds within the Burrunan dolphin repertoire, this species stands out among *Tursiops* spp., or burst-pulse sound contribution to delphinid vocal repertoire is under-represented in previous studies. Behavioural contexts in which burst-pulse sounds have been recorded include bouts of aggression [92], mate guarding and reproduction [91,93], and social interaction [90] in common bottlenose dolphins.

We note the lack of a naming convention for burst-pulse sounds in the literature. While a quantitative system based on acoustic (spectrographic) features might be more robust, the literature largely presents onomatopoeic names [26]. The risk is that the same signal sounds different to different researchers and so is assigned different class names in different studies [26]. Here, we present both quantitative and aural features, in an attempt to compare the Burrunan burst-pulse sounds with those reported for other *Tursiops* spp. in the literature. While we found differences, further study will be required to understand the degree of and drivers for these differences.

#### 4.2.1. Barks

The bark was the most common burst-pulse sound in the Burrunan dolphin repertoire. Barks possessed the smallest standard deviations of all vocal signal classes measured, indicating their consistency and likely stereotypy. They resemble the low-frequency narrow-band (LFN) sounds described by Schultz *et al.* [90] and Gridley *et al.* [42] and the gulps outlined in Luís *et al.* [94], as discussed in reviews of the literature [26]. The Burrunan barks resemble the LFNs of Schultz *et al.* [90] in that they exhibit both a trill-like and hoot-like sound quality (when above and below 1 kHz, respectively); however, the duration and frequency range of Burrunan dolphin barks more closely match the LFNs of Gridley *et al.* [42] and the gulps of Luís *et al.* [94]. As in the case of all three of these descriptions, Burrunan dolphin barks are emitted in ‘trains’ of multiple pulsed signals. Gridley *et al.* [42] associated LFNs with several behaviours, dependent on the accompanying vocalizations; LFNs paired with whistles related to feeding and social behaviours, while LFNs within bray calls related to aggression. Surface social behaviours were also observed by Schultz *et al.* [90] when recording LFNs, though this also involved physical contact between individuals and was therefore associated with mating.

#### 4.2.2. Squeaks

Burrunan dolphin squeaks visually resemble the burst-pulse sound included in the bray sequence of *Tursiops* spp. described by Janik [95]. Further, Burrunan dolphin squeaks share spectrographic characteristics, including duration and frequency range, of the ‘squeak’ element of the common bottlenose dolphin bray sequence identified by Luís *et al.* [94]. Additionally, the Luís *et al.* [94] squeak is described by authors as sounding tonal despite being pulsed, as is the case in Burrunan dolphin squeaks. In both studies, bray sequences are identified as repeated patterns of varying signal types and classes, associated with feeding [95]. In this way, the presence of squeaks within acoustic recordings of Burrunan dolphins may be able to be used to indicate feeding activity.

#### 4.2.3. Creaks

Signals similar to Burrunan dolphin creaks are referred to by different onomatopoeic names in the literature, such as ‘squeaky gates’ and ‘screeches’ [93,96]. The individual pulses within the creak trains are reminiscent of the ‘pops’ associated with male mate guarding in *Tursiops* spp. [97–99] and are therefore believed to be linked to aggressive social and reproductive behaviours. However, Burrunan dolphin creaks display much smaller interval between the individual pulses in the train and produce a very different aural quality, as seen in common bottlenose dolphins [38]. The creaks of Luís *et al.* [38] were not associated with a given behaviour, though authors highlighted that creaks were previously described as a class of echolocation associated with feeding [100]. The variation in bandwidth of Burrunan dolphin creaks is probably due to the directionality of these signals and varying angles of incidence on our hydrophone [37,40]. Future study of creaks should record at higher sample rates to capture the full bandwidth.

#### 4.2.4. Moans

Burrunan dolphin moans resemble other previously described modulated tonal contours with many overtones [43,101]. These probably result after NFFT, given the very small IPI between individual pulses [102]. Moans are very rarely described in the literature, possibly owing to the likelihood of masking by similarly modulated vessel noise [43] as well as their lack of association with observable, surface-active behaviours [101]. In human-managed individuals, moans were recorded prior to an enrichment event, such as feeding or human interaction [101], and accompanied additional behaviours such as bubble streaming or spy-hopping, indicating that the vocalization may be associated with an anticipated behaviour change.

### 4.3. Burrunan dolphin populations

The PPB and GL populations exhibited statistically significant differences in whistle repertoires for five of the eight fundamental frequency parameters (*F*_start_, *F*_end_, duration, presence of overtones and number of steps). The burst-pulse sound repertoires of the two Burrunan dolphin populations also exhibited statistically significant differences in all measured parameters. As stated above, high levels of stereotypy within whistle repertoire have been linked to acoustically complex environments [84,85]. Further, it has been suggested that number of steps within dolphin whistle contours may be unique at the species or population level and may relate to the complexity of information encoded within the whistle [103]. Greater disparity in the standard deviations in all measured parameters in both the whistles and burst-pulse sound repertoires were observed in the GL population, which may indicate the vocal repertoire is more conserved in PPB than in GL, and the vocal repertoire of GL may exhibit greater variation between individuals. The high conservation of the PPB repertoire may be related to the higher likelihood of interaction with other cetaceans (conspecifics and non-conspecifics) within the PPB environment.

Genetic evidence confirms some members of the GL population travel between the Victorian coastline and Tasmania [104], an island-state off the southern coast of mainland Australia connected via a body of water called Bass Strait, and thus these animals may interact with other cetacean species during their transit. Stranding records indicate at least 31 cetacean species visit the pelagic waters of Victoria [105]. However, seasonal variability in GL population size confirms the majority of the population is resident to the shallower GL habitat, and so, only a small subset of the population will encounter other cetaceans in Bass Strait [2]. This prevents most of the GL population interacting with other vocalizing cetaceans, allowing the GL resident vocal repertoire to evolve unchecked. Conversely, cetacean species such as humpback whales (*Megaptera novaeangliae*) and southern right whales (*Eubalaena australis*) visit PPB [105,106] as a place of refuge on their migration along Australian coastlines. Additionally, the PPB Burrunan dolphin population shares its habitat with a small resident population of short-beaked common dolphins (*Delphinus delphis*) [107]. Sympatric bottlenose and common (*Delphinus* spp.) populations have been demonstrated to produce significantly different whistle repertoires [55]. The entire Burrunan dolphin population in PPB is, therefore, more likely exposed to acoustic repertoires of resident and visiting non-conspecifics, converging to a less variable and more stereotypical whistle repertoire than that recorded in GL.

Moreover, whistle and burst-pulse sound features may be driven by the local environment, including its ambient noise and sound propagation conditions. PPB is an on-average 13 m deep bay that encompasses Australia’s busiest commercial port, whereas GL is a 4 m deep estuarine and wetland system frequented only by small boats. Ambient noise has been shown to affect frequency parameters, duration and repetitions of whistles in *Tursiops* spp. [84–86,108,109]. The sound propagation conditions of these shallow-water environments may also affect whistle features, as energy at low frequencies is potentially absorbed over shorter ranges in shallow water (depending on seafloor properties) [78]. Environmental factors, such as temperature gradients, currents, depth, obstacles in the sound path and bottom characteristics have been demonstrated to affect the acoustic space of *Tursiops* signals [110].

Of note, significantly more acoustic recordings were taken in GL (approx. 4 and 16 h of recording in PPB and GL, respectively). However, normalized whistle and burst-pulse sound counts indicated no significant difference in representation of population-specific vocal signals within the dataset. Further, it is possible that not all population demographics and functional behaviours may be represented in this dataset. Given the requirement for the research vessel to be stationary and switched off during recording, strong bias towards recording vocalizations emitted during stationary behaviours, such as foraging and milling/resting, will have been recorded in this dataset. Some functional behaviours, such as group cohesion required for travel, may not have had their associated vocalizations captured. This repertoire-specific study also does not account for which individual dolphins and/or demographics had been captured in dataset, an area that needs to be further explored when assessing population and/or individual based signals, for example, through the investigation of signature whistles.

### 4.4. Comparison with other *Tursiops* populations

The Burrunan whistle repertoire was compared with the repertoire of populations of both common bottlenose dolphin (*Tursiops truncatus*) and Indo-Pacific bottlenose dolphin (*Tursiops aduncus*) recorded on the west coast (Fremantle [71,74], Bunbury [76,77] and Shark Bay [30,76]) and the east coast of Australia (Moreton Bay [76] and Byron Bay [76]). All of these represent resident coastal *Tursiops* populations of similar population structure, ecological habitats and selective pressures to that of the two Burrunan dolphin populations in Victoria (PPB and GL), in their shallow-water environments, their proximity to populated coastlines and the presences of anthropogenic threats.

The Burrunan dolphin whistle repertoire was most similar in class composition to that of the resident Indo-Pacific bottlenose dolphin population in Bunbury, Western Australia [76,77]. These dolphin populations exhibit similar population structures, possessing complex social dynamics with matriarchal pod structuring centred around calving [2,111]. Further, both the Bunbury dolphins and Burrunan dolphins share similarities in habitat composition, with each population inhabiting combined brackish estuarine and salty marine environments [2,111]. Additionally, both populations are subject to high levels of human interaction, with Victoria’s swim-with-dolphin tours [10] and Bunbury’s dolphin feeding experiences [112], when compared with the other populations analysed. As in the case of behavioural changes seen in the Burrunan dolphin in response to eco-tour [17] or recreational [11] vessel presence, the impacts of anthropogenic activities on the Bunbury dolphin population are demonstrated in behavioural changes away from natural states, such as reduced socializing, potentially leading to reduced reproductive success [113]. The presence of humans [114] or anthropogenic noise [109,115] has been directly connected to manipulations in dolphin acoustic repertoire and may provide an explanation as to why these populations exhibit the most similar whistle repertoires despite residing on opposite sides of the continent.

As the *Tursiops* genus is distributed across the globe [116], their acoustic ecologies are studied in a great diversity of functional and anthropogenically impacted habitats. To grow our joint understanding of dolphin acoustic communication, a collaborative, standardized research effort is required. Given the small quantities of the complex whistle classes (convex, concave and sine) in *Tursiops* spp. whistle repertoires, grouping of these calls into a single ‘complex’ class as undertaken in other studies [74,76,77] can better facilitate comparison of repertoire composition. The pooled ‘complex’ whistle contour comprised 40.97% of Burrunan dolphin repertoire (figure 10), providing greater sample for comparison with other populations. However, finer class differentiation carrying more specific information will be missed. Further, the whistle repertoire datasets used for comparison with the Burrunan dolphin repertoire comprised only whistle class proportion data. In this way, biases associated with data collection methodologies, including unequal sample sizes for populations or discrepancies in recording length, may not be accounted for in these analyses. Vocalization datasets and analysis methods must be comparable and ideally open source for informed interpretation so we may understand the drivers behind the differences in vocalization characteristics and repertoire composition.

Significant differences have been observed between *Tursiops* spp. populations globally, believed to be influenced by geographic separation and social isolation [117,118], localized environmental conditions [77,91] or a combination of these factors [34]. Geographical variation in the acoustic characteristics of vocal signals has been investigated for numerous delphinid species, in particular in the case of the *Tursiops* genus [27,30,75,76,84,119,120]. Alternatively, there is also evidence that despite the geographical isolation of allopatric populations, significant similarities exist in the vocal repertoires of *Tursiops* spp. around the world [27,30]. Some of this similarity may be due to similar morphologies limiting the diversity of signals produced. Additionally, the highly conserved vocal signals may have convergently evolved together with critical biological processes or survival strategies, such as facilitating resource sharing in non-allopatric populations [27].

### 4.5. Significance

Given the recent identification of the Burrunan dolphin species and its critically endangered conservation status, there is still much to be explored and understood. Here we provided a first assessment of the whistle and burst-pulse sound vocal characteristics for the Burrunan dolphin. Both the PPB and GL Burrunan dolphin populations produce the same vocalization types and classes, though differences in repertoire proportion by sound class and specific acoustic features were found. Differences in functional habitat usage, ambient noise and sound propagation conditions may all contribute to the variation in whistle and burst-pulse sound characteristics between the two geographically isolated Burrunan dolphin populations and across *Tursiops* species. Given the Burrunan dolphin is a top-order predator within both the PPB and GL environments, the species is a key indicator for the overall health and productivity of these ecologically and economically significant locations. Baseline understanding of the functional behaviours of this species, including its acoustic behaviours, is needed for effective, non-invasive (passive acoustic) monitoring and ultimately conservation management.

## Data Availability

Descriptive statistics for all whistle and burst-pulse sound classes can be found in the electronic supplementary material [121]. Raw data may be accessed by request via contacting the corresponding author at research@marinemammal.org.au.
